# Closing the Loop: Solid Oxide Fuel and Electrolysis Cells Materials for a Net-Zero Economy

**DOI:** 10.3390/ma17246113

**Published:** 2024-12-13

**Authors:** Mirela Dragan

**Affiliations:** National Research and Development Institute for Non-Ferrous and Rare Metals—IMNR, 102 Biruintei Blvd., 077145 Pantelimon, Ilfov, Romania; mirelad.dragan@gmail.com

**Keywords:** solid oxide fuel cells (SOFCs), solid oxide electrolyzers (SOECs), circular economy, recycling, critical raw materials, sustainable energy

## Abstract

Solid oxide fuel cells (SOFCs) and solid oxide electrolyzer cells (SOECs) represent a promising clean energy solution. In the case of SOFCs, they offer efficiency and minimal to zero CO_2_ emissions when used to convert chemical energy into electricity. When SOFC systems are operated in regenerative mode for water electrolysis, the SOFCs become solid oxide electrolyzer cells (SOECs). The problem with these systems is the supply and availability of raw materials for SOFC and SOEC components. This raises significant economic challenges and has an impact on the price and scalability of these technologies. Recycling the materials that make up these systems can alleviate these economic challenges by reducing dependence on the supply of raw materials and reducing overall costs. From this point of view, this work is a perspective analysis and examines the current research on the recycling of SOFC and SOEC materials, highlighting the potential paths towards a circular economy. The existing literature on different approaches to recycling the key materials for components of SOFCs and SOECs is important. Mechanical separation techniques to isolate these components, along with potential strategies like chemical leaching or hydrometallurgical and material characterization, to ensure the quality of recycled materials for reuse in new SOFCs and SOECs are important as well. By evaluating the efficiency of various methods and the quality of recovered materials, this study aims to provide valuable insights for advancing sustainable and economically viable SOFC and SOEC technologies within a net-zero economic framework.

## 1. Introduction

Global energy consumption is expected to increase substantially in the coming decades [1,2]. What drives this forecast is based simultaneously on the technological and industrial progress along with the growth of population. Unquestionably, for many decades, fossil fuels were the primary source of energy. It provided most of the energy needed around the world. But nowadays, the fossil fuels which are resources subject to limitations, are considered to be disadvantageous for society’s standards. They have expensive costs for their extraction and have an equally costly impact on the environment and the economy [3]. The impact on the environment as a result of fossil fuel consumption is a major concern these days. This is because the burning of fossil fuels releases greenhouse gases into the atmosphere, contributing to climate change [4]. On the other hand, the extraction of fossil fuels often involves technologies such as mining and drilling that are considered destructive to the soil. These technologies can lead to the destruction of environment, contamination of water or pollution of air. Comparing consumption sectors, it appears that this industry has the largest share of energy consumption, with the transport and residential sectors following closely behind. Understanding these consumption patterns is a necessary condition when trying to develop effective energy policies and strategies. The Global Greenhouse Gas Reference Network (acronym GGGRN) tracks changes in major anthropogenic sources of greenhouse gases. These gases include carbon dioxide CO_2_, methane CH_4_, and nitrous oxide N_2_O [5]. It has been shown that worldwide, on average, each person is responsible for the emission of approximately 4.7 metric tons of CO_2_, through their energy-related activities [6]. In general, the consequences of climate change are numerous and severe. These include droughts, fires, floods, reduced availability of drinking water sources, melting polar ice, and storms [7]. The impact of climate change creates various issues for biodiversity. Many species struggle to adapt to the developing changes in environmental conditions, and not all species succeed. This failure leads to declining populations and, in some cases, even extinction. The projected decline in biodiversity will be accompanied by a significant impact on functionality of the ecosystem, security of food and, generally, on the quality of human life at a large scale.

Nowadays, addressing the increasing demand for energy in our modern society goes hand-in-hand with efforts to mitigate the impacts of climate change. This issue is complex and requires dedicated efforts to ensure the long-term sustainability of our planet. In search of a solution to the current energy situation by taking care of climate change, meetings at the international level have been organized and several global agreements have been signed. In this regard, the Rio Convention at the United Nations Conference on Environment and Development in 1992 should be mentioned. This event marked a significant step towards international cooperation in environmental issues. The Kyoto Protocol is also worth noting. This was negotiated in December 1997, and it is the first legally binding international agreement which aims to reduce greenhouse gas emissions. The Paris Agreement also had a significant impact, and it can be defined as a major milestone in global climate policy. It was signed in 2015, and it aims to limit global warming to less than 2 °C compared to pre-industrial times [8,9,10]. What makes it more impactful than the Kyoto Protocol is the Paris Agreement includes commitments from both developed and developing countries. This agreement also emphasizes the importance of technology adaptation, financing and transfer to support climate action.

Looking from this perspective, solid oxide fuel cells (acronym SOFCs) and solid oxide electrolysis cells (acronym SOECs) are expected to effectively make a significant impact on the future energy landscape. These technologies can provide energy sources that are both cleaner and more efficient, and thus are environmentally friendly energy devices. This is essential as the entire world strives for a sustainable energy future. SOFCs and SOECs offer a number of strong advantages making them far superior to conventional energy technologies. SOFCs and SOECs offer significant potential since they have high efficiency and produce low emissions when working [11]. This year, the U.S. Department of Energy’s (DOE) Office of Fossil Energy and Carbon Management (FECM) announced up to $4 million in federal funding to make clean hydrogen a more available and affordable fuel for electricity generation, industrial decarbonization, and transportation [12]. Counted as an advantage for these devices, it is also opens the possibility of using a variety of fuels. These fuels include hydrogen, natural gas and biofuels. Additionally, there is the option of integrating these technologies with renewable energy sources such as solar and wind. In this manner, it can provide reliable and sustainable energy options.

**Figure 1:**
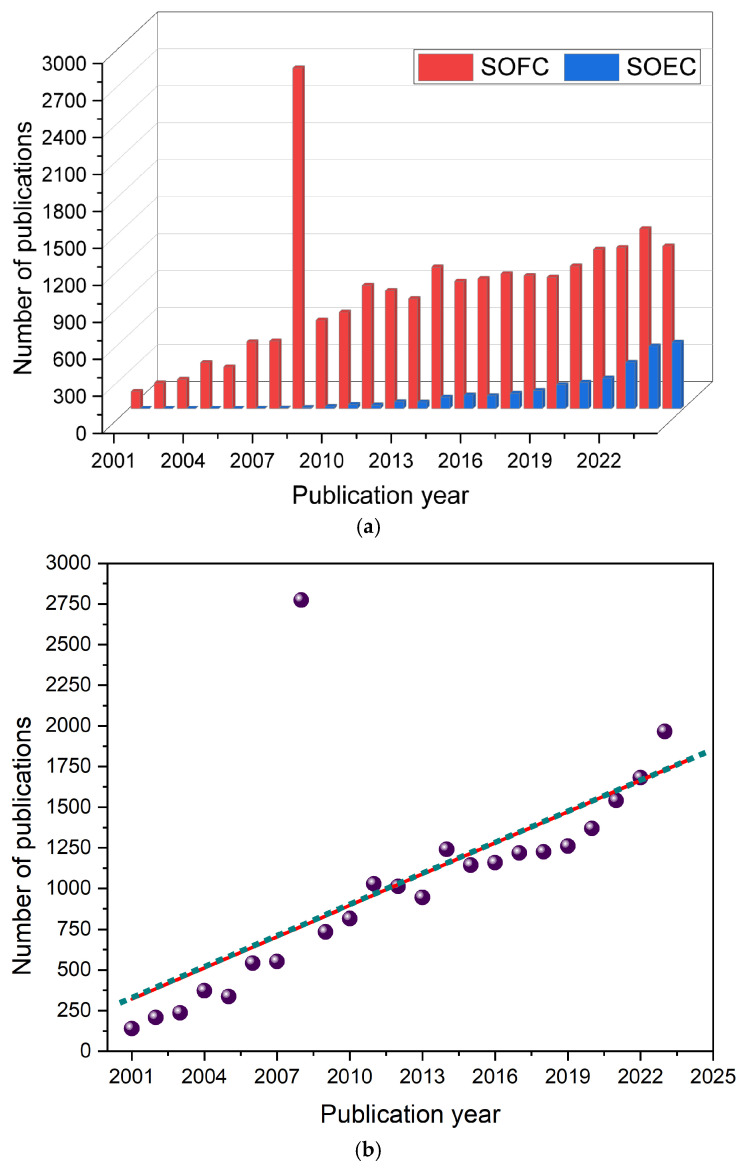
(**a**) Comparing the number of publications related to SOFCs and SOECs from 2001 to 2023 [13]. The red color represents SOFCs publications, while the blue color represents SOECs publications. (**b**) The cumulative SOFC and SOEC number of publications over time, from 2001 to 2023. The publications are represented by a purple color and the trend is visualized using a linear regression line.

The research activities through publications in this particular field were surveyed. The count of publications in specific journals based on various keywords for this topic was gathered and visual representations of the data were created. Both Figure 1a,b collectively provide insight into the research landscape. Figure 1a highlights the general increase in publications, while Figure 1b provides a detailed comparison of the research in each field separately. These visual presentations suggest that while research community interest in SOFC has steadily increased, the specific area of SOEC is gaining momentum. This might potentially indicate shifts in emerging technologies. On the *y*-axis is presented the number of publications, while on the *x*-axis is presented the year of publication. There is a clear upward trajectory in the number of publications related to SOFC and SOEC technologies from 2001 to 2023 [13]. Both SOFC and SOEC research activities show sustained growth. This counts as an indicator for continued interest in the field and a promising future for these technologies in various applications. From the data it can be calculated that the average annual growth rate for SOFCs is 14.97% and the average annual growth rate for SOECs is 12.59%. SOFCs have been a more dominant research area compared to SOECs. The global push for clean energy solutions was intensifying. This is probably due to the fact that many nations began setting ambitious goals for reducing carbon emissions which boosted research in fuel cell technologies. The increasing trend in SOEC publications indicates a growing recognition of its potential driven by advances in renewable energy and hydrogen production technologies. The year of 2008 is notable for an exceptionally high number of SOFC publications compared to the surrounding years showing that perhaps fast-moving research areas had dedicated funding available. However, there is a noticeable decline in SOFC-related publications in subsequent years, likely due to several factors. This possibly indicates that around this time, interest or supportive policies started shifting toward other clean energy technologies such as lithium-ion batteries, solar photovoltaics and wind energy.

Nowadays, sustained efforts in research and development that focus on improving the performance, durability and profitability of SOFCs and SOECs can be observed. From this point of view, advances in material science, manufacturing processes and systems design also lead to advances in the field of SOFCs and SOECs. Through this advancement, it is possible to bring these technologies closer to commercial viability. However, like many of the advanced technologies of these days, the manufacturing of SOFCs and SOECs depends on critical raw materials (acronym CRMs). Therefore, it is necessary and essential to have a recycling strategy. This has a crucial role in achieving a net-zero economy by reducing waste, conserving resources and minimizing environmental impact.

In the context of SOFC and SOEC recycling, life cycle management of the materials and components used in these technologies is essential. Current recycling practices for SOFC and SOEC materials should involve the recovery and reuse of valuable metals, ceramics, and other materials.

The Critical Raw Materials Act Regulation outlines a number of activities in the context of ensuring the European Union’s (acronym EU) long-term supply of critical raw materials [14]. Also, the Critical Materials Institute (acronym CMI) of the United States Department of Energy (acronym DOE), focuses on the entire life cycle of materials, from their extraction to their use and disposal. This is to explore how to reduce the impact of supply chain disruptions and price fluctuations [15].

The classification of critical raw materials generally involves factors such as economic importance and the associated supply risk. In 2020, the European Commission published its revised list of critical raw materials [16].

**Figure 2:**
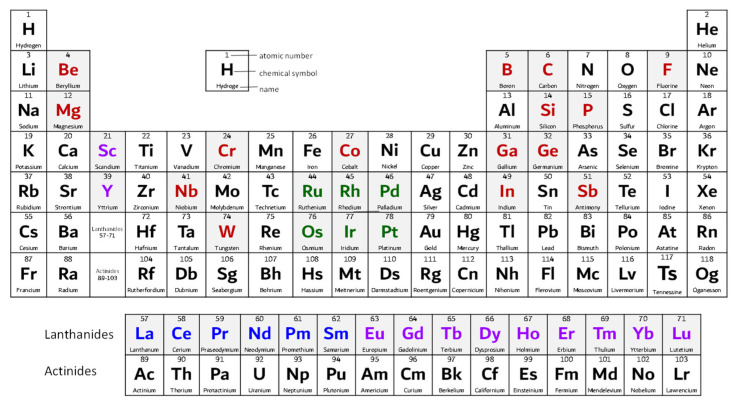
Materials considered critical raw materials are marked in different colors.

Materials considered as critical raw materials are grouped, Figure 2, and marked in four colors as follows: (i) in red, (ii) in blue, (iii) in magenta and (iv) in green. The term ‘rare earth elements’ (acronym REEs) is a collective name for the 15 elements in the lanthanide group and they are categorized into rare earth elements-light (acronym LREE)—marked in blue; and rare earth elements-heavy (acronym HREE)—marked in magenta. Marked in green are the six precious metals, platinum group metals (acronym PGMs), which include ruthenium, rhodium, palladium, osmium, iridium and platinum [17]. Due to some of their similar properties, scandium and yttrium are also often considered as rare earth elements [18,19,20]. The rest of the critical raw materials, not classified as rare earth elements or precious metals, are marked in red.

The International Energy Agency (acronym IEA) predicts that the global market for essential products of advancing net-zero transitions is prognosticated to triple by 2030 [21]. In order to meet the growing demand of critical raw materials, as well as raw materials in general, it is imperative to seek out solutions. In this line of action, increasing recycling will be an important step towards a more sustainable materials usage. Implementing effective recycling strategies at the end-of-life phase for each material, especially for critical materials, can significantly mitigate environmental harm [22,23].

Recycling materials used in SOFCs and SOECs, such as metals and ceramics, would contribute to resource conservation by reducing the need for new and eventually raw material extraction including critical material extraction. The United Nations Industrial Development Organization (acronym UNIDO) predicts the demand for raw materials used in the manufacturing sector will double by the year of 2050, reaching ~180 billion tons [24].

The European Parliamentary Research Service (acronym EPRS) concluded a circular economy reduces waste by employing methods such as reusing, repairing, refurbishing, and recycling materials and products already used [25]. In a linear economy, resources are extracted from the underground and transformed into products. After the products have been used, they are disposed as waste.

Implementing circular practices for the materials used in the energy sector involves several challenges. These should focus on the needs for technological innovation, regulatory support, and stakeholder collaboration However, the benefits of a circular economy are substantial and include reduced resource consumption, lower greenhouse gas emissions, and enhanced economic resilience.

Examples of circular economy initiatives in other industries, such as electronics, automotive, and construction, provide valuable insights and lessons on the materials used in the energy sector [26,27]. These successful recycling initiatives have demonstrated the potential to reduce the environmental footprint of these technologies, drive sustainability and create new business opportunities.

This integration of the circular economy with low-carbon technologies fosters a more resilient and sustainable future by tackling both the pressing issues of climate change and the long-term needs of the population at global level.

Nonetheless, the recycling process for SOFC and SOEC materials encounters various ongoing difficulties. These include the complexity of material separation, the presence of contaminants, and the need for efficient and cost-effective recycling processes. This work aims to find efforts in this area of recycling of materials for SOFC and SOEC components. It is useful to identify research gaps, in particular, for assessing the future availability of materials used primarily for the manufacturing of SOFC and SOEC components. This will help to secure a resilient, sustainable and ethical supply of critical raw materials for SOFC and SOEC components and the transition to a net-zero economy.

## 2. Solid Oxide Fuel Cells (SOFCs)

Typically, a fuel cell consists of three essential active layers as follows: the anode, which is the fuel electrode, the cathode, which is the oxidant electrode, and the electrolyte layer situated between them. The electro-chemical phenomena occur at the anode and cathode generating electricity. This electricity can be used to perform beneficial functions, such as powering electrical devices or vehicles, or supplying electricity for the grid.

There are numerous conditions under which fuel cells can operate. Different operating conditions relate, for example, to the fuel type or operating temperature. Additionally, fuel cells can have in their construction different electrolyte material type that allows them to be classified. Conventionally, fuel cells are categorized based on the type of electrolyte material used in them. The fuel cell types are as follows: polymer electrolyte membrane fuel cells (acronym PEMFC), phosphoric acid fuel cells (acronym PAFC), alkaline fuel cells (acronym AFC), molten carbonate fuel cells (acronym MCFC), solid oxide fuel cells (acronym SOFC), and direct-methanol fuel cells (acronym DMFC).

The cell name is determined by the type of electrolyte, but it is also differentiated by the operating temperature for each fuel cell.

**Figure 3:**
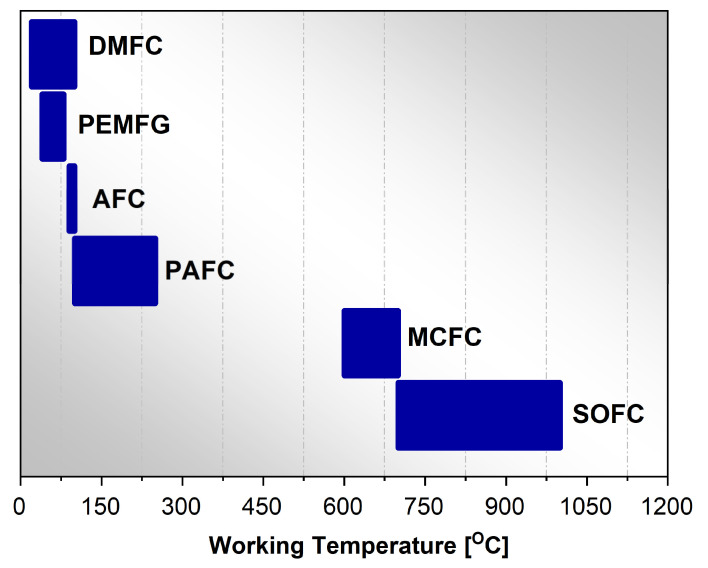
Operating temperatures for all fuel cells.

Figure 3, a horizontal bar chart, illustrates the operating temperature ranges for different types of fuel cells. The *x*-axis displays the operating temperature in degrees Celsius (C), which ranges from 0 to 1200 °C. The *y*-axis has no specific units, but for clarity in visualization, they were used separately between the types of cells. The *y*-axis lists the different categories of fuel cells: DMFC, PEMFG, AFC, PAFC, MCFC, and SOFC. Each type of fuel cell is represented by a horizontal bar, indicating its operating temperature range.

The wide range of operating temperatures implies that different materials are required for each fuel cell type. This is to withstand the respective thermal conditions. Each type of fuel cell offers specific advantages as well as certain limitations. Their utilization depends on the specific application requirements such as operating conditions, and fuel availability. Research for materials used in the construction of fuel cells is a continuous activity in various research groups.

In the matter of the SOFCs, these operate at high temperatures, above 800 °C, and they use a solid ceramic electrolyte [28]. Owing to the highest operating temperature range, SOFCs are ideal for high-temperature industrial processes, as well as integrated heat and power (acronym CHP) systems [29]. The SOFCs are suitable in stationary power generation, such as in residential, commercial and industrial settings as well as generating remote off-grid power and providing onboard power for vehicles.

**Figure 4:**
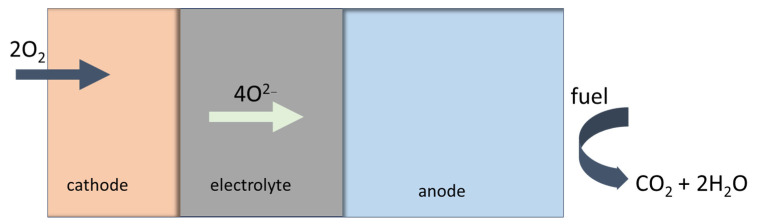
Working principle for Solid Oxide Fuel Cell (SOFC).

Fuel like hydrogen, natural gas or biogas, is supplied to the anode side. Once the anode is reached, the fuel reacts with the oxygen ions from the electrolyte generating water vapor (H_2_O), carbon dioxide (CO_2_) and releasing electrons see Figure 4. The anode itself is catalytically active, and it is promoting the electrochemical reactions. At the cathode, the oxygen from the air or from an oxygen-containing gas is reacting with the electrons and oxygen ions. The oxygen ions are conducted through an electrolyte to form oxygen gas (O^2−^). The cathode material acts as a catalyst enhancing the oxygen reduction reaction. The solid electrolyte allows the migration of oxygen ions from the cathode to the anode.

Because of the high temperatures involved during the operation, light hydrocarbon fuels such as methane, propane or butane have the potential to internally reform within the anode. But SOFCs also have the alternative of being fueled by externally reforming, using denser hydrocarbons or biofuels. Additionally, SOFCs have the alternative of solid fuels like coal and biomass with potential for gasification, resulting in syngas. This is suitable for generating power in SOFCs within integrated gasification fuel cell power cycles.

## 3. SOECs: Components and Operation

Electrochemical water splitting is considered one of the most environmentally friendly techniques for producing hydrogen because it does not contribute to carbon emissions.

The reaction for the electrolysis of water is as follows:

H_2_O ⟶ H_2_ +1/2 O_2_ (1)

At room temperature, 25 °C, and atmospheric pressure, the change in enthalpy of this reaction is ΔH = +286 kJmol^−1^. The reaction is increasingly endothermic with increasing temperature.

At present, there are four main types of electrolysis technologies: alkaline water electrolysis (acronym AWE), proton exchange membrane electrolysis (acronym PEM), solid oxide electrolysis (SOEC), and anion exchange membrane (acronym AEM) electrolysis.

**Figure 5:**
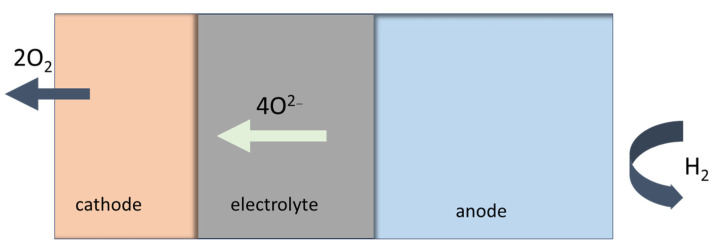
Working principle for Solid Oxide Electrolysis Cell (SOEC).

The basic structure and operating principles of SOECs, Figure 5, are the same as those of a SOFCs, described above. Nevertheless, in terms of how it works, a SOEC operates essentially in the opposite manner compared to a SOFC. Instead of utilizing hydrogen to generate electricity, a SOEC make use of electricity to produce hydrogen. The reactions for one mole of water are shown below. The oxidation of water occurs at the anode, oxygen evolution reaction (acronym OER), and the reduction of water, hydrogen evolution reaction (acronym HER), occurs at the cathode.

Anode: 2 O^2−^ → O_2_ + 4 e^−^ (2)

Cathode: H_2_O + 2 e^−^ → H_2_ + O^2−^ (3)

Overall Reaction: 2 H_2_O → 2 H_2_ + O_2_ (4)

## 4. Relevant Critical Raw Materials in SOFCs and SOECs

The choice of material is essential to the overall performance, durability, and efficiency of SOFC and SOEC systems. The coefficient of thermal expansion (CTE), mechanical stability, oxygen ion conductivity, electrical conductivity, and chemical stability are important considerations when designing SOFC and SOEC systems because they influence the choice of materials for the component parts.

However, research in materials science has concluded that a limited number of materials meet these stringent requirements, particularly those related to oxygen ion conductivity, limiting the choice of viable materials for these applications. Regarding the CTE, since there are different layers in SOFCs and SOECs, it must be comparable to prevent thermal cracking and delamination. The electrolyte, electrodes and interconnects must expand and contract at similar rates during heating and cooling cycles. Any mismatch in the thermal expansion behavior of the materials can lead to mechanical failure through cracking and delamination. Mechanical instability and delamination are reducing the cell life by leading to more frequent replacements, and consequently causing higher operational costs.

Another basic requirement for SOFCs and SOECs is the ability to conduct oxygen ions through the electrolyte. The materials for the electrolyte must have a high conductivity of oxygen ions to ensure efficient electrochemical reactions inside the cells. This property is crucial to achieve high power densities and low internal resistances.

For optimal performance, electrodes must exhibit both high electronic and ionic conductivity to facilitate the necessary electrochemical reactions. Materials such as nickel-based cermets for anodes and perovskites for cathodes are used since these requirements are met. The materials must withstand the harsh chemical environments of SOFCs and SOECs, such as exposure to oxygen, hydrogen or other fuels at high temperatures, without degrading over time. In order to avoid reactions that might result in decreased performance or material damage, chemical stability is essential.

**Figure 6:**
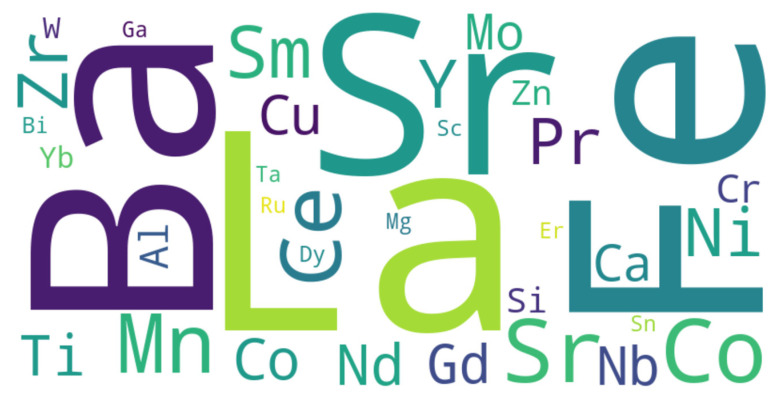
This visual summary highlights the key chemical elements in the context of the development and optimization of SOFCs and SOECs.

Once again, given these requirements, one can find that there are relatively few key materials that are suitable for these applications. In Figure 6, each element’s relative importance and frequency of use in SOFC and SOEC materials are indicated by the size and color of its symbol in the word cloud. Elements that are essential to these technologies are represented by larger, more noticeable symbols.

The word cloud features the chemical elements, collected from the literature, commonly associated with materials used in SOFCs and SOECs [30,31,32,33,34,35]. Elements that are larger and more central, such as La, Sr, Co, Ba, and Ni, indicate their more frequent use in materials from the SOFC and SOEC components, which means that they are also important. Each element contributes distinct physico-chemical properties that are critical to the performance of various components within SOFCs and SOECs, such as the cathode, anode, and electrolyte.

The demand of raw material supply for promising materials in the field of SOFC and SOEC materials research may be predicted. This can be carried out by making use of data demonstrating the dependencies between chemical elements and their combinations in material compositions. Exploratory data analysis of compiling the literature to identify key chemical elements and their pairs within SOFC and SOEC materials help us to understand their co-occurrence. The combinations in which the chemical elements appear most frequently can be seen, as well as in how many combinations of materials they appear.

**Figure 7:**
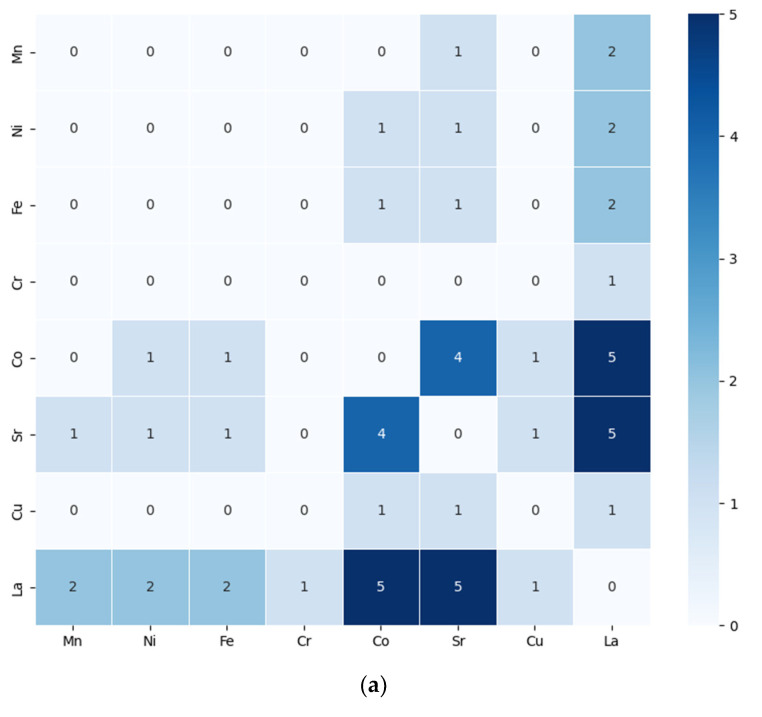

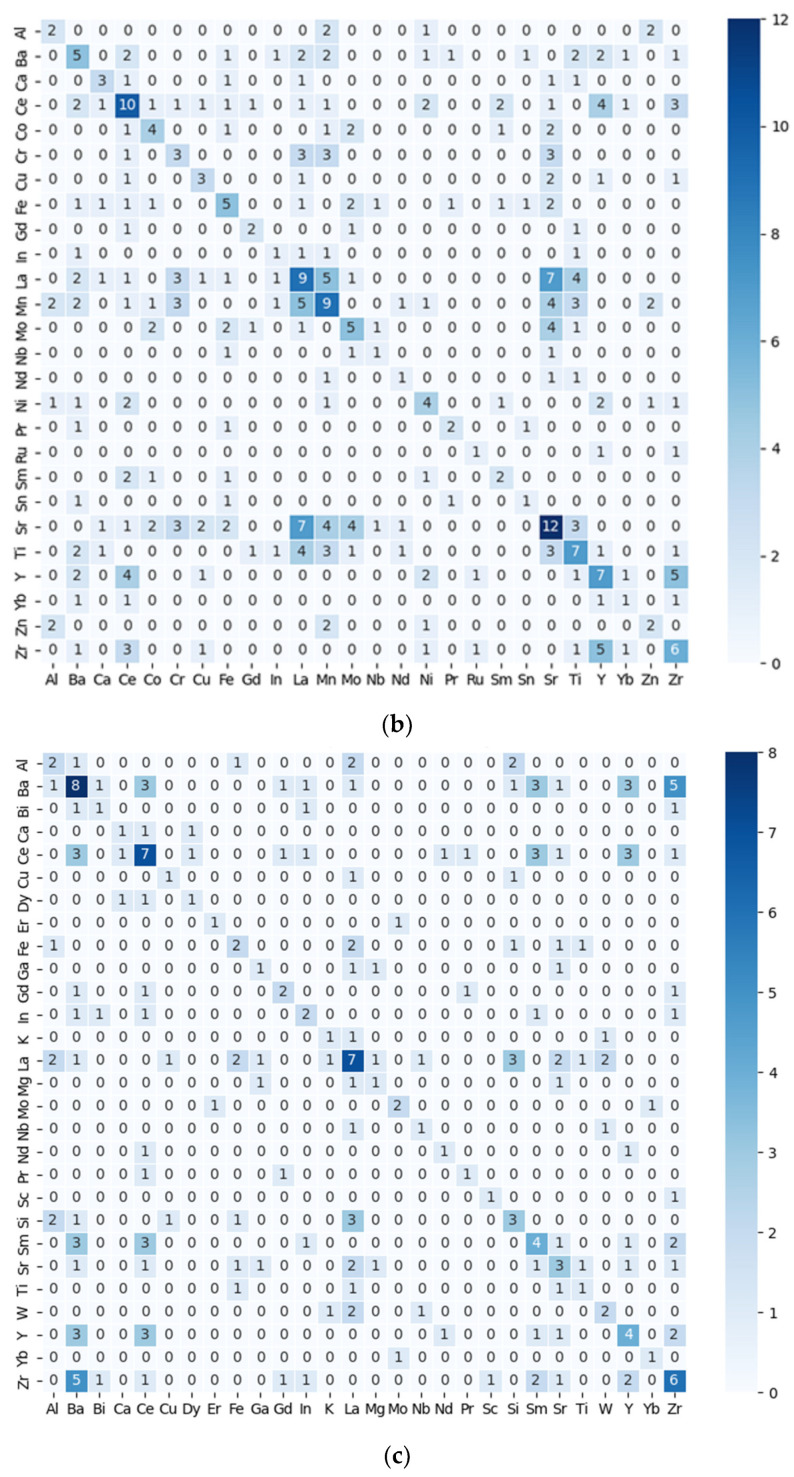
(**a**) Heatmaps of co-occurrence SOFCs and SOECs cathode materials; (**b**) heatmaps of co-occurrence SOFCs and SOECs anode materials; and (**a**,**c**) heatmaps of co-occurrence SOFCs and SOECs electrolyte materials.

For a visual way to summarize the findings and main components in this work, heatmaps are used, Figure 7. These are a two-dimensional data visualization method that uses color to show the magnitude of each individual value in a dataset. On the other hand, the heatmaps of the co-occurrence matrix between various pairs would show how frequently two elements occur together.

In the heatmap, the value of 5 for the combination of La, Sr, and Co means that these three elements were associated together five times in the dataset being visualized, as they were found together in a set of materials. La, Sr, and Co are elements commonly involved in perovskite oxides for SOFCs/SOECs materials. The value of 4 for the combination of Sr and Co reflects their frequent pairing in oxides for SOFCs/SOECs materials contexts. A moderate frequency of co-occurrence is the combination of La, Fe, Ni, and Mn. The lowest non-zero frequency of co-occurrence, 1, in the heatmap, indicates a rare association between the two elements compared to other combinations with higher values.

High co-occurrences of Sr and Y, with a value of 12, and Zr and Al suggest these elements are commonly paired in the materials. There is a moderate co-occurrence with a value of 5 to 7 indicating frequent but not dominant associations between the marked elements. The low co-occurrences with a value of 1 or 2, show rare instances of element combinations, possibly indicating niche applications.

Ba and Zr with a value of 8, La and Mn with a value of 7, Yb and Zr with a value of 5, and Ca and Zr with a value of 4, are pairs of elements frequently found together, suggesting that they have a strong association in the materials. Pairs with co-occurrence values between 1 and 3, such as Nd and Sr with a value of 3, Pr and Sr with a value of 3, and La and Fe with a value of 3, are elements that possibly represent niche combinations.

Overall, this co-occurrence heatmap provides a visual summary of how different elements are associated in SOFCs/SOECs research, helping to highlight potential areas of exploration in material design or synthesis. Understanding which elements frequently co-occur can guide decision-making in future material development efforts.

These elements are engineered into complex materials that have synergistic effects, increasing the overall efficiency and durability of SOFC and SOEC devices, rather than being selected solely for their individual qualities.

In this regard, a prominent pair which indicate a strong association is between La and Sr. Lanthanum (La) and strontium (Sr) are often paired in perovskite-type cathodes, such as in La_1−X_Sr_X_MnO_3_ (LSM) or La_1−X_Sr_X_CoO_3_ (LSC).

The structure of LaCoO_3_ and LaMnO_3_ is derived from the cubic ABO_3_ perovskite. The replacement of lanthanum with strontium leads to the generation of oxygen vacancies, which compensates for the difference in ionic charge between Sr^2^⁺ and La^3^⁺.

The LSM/YSZ cathode has proved to be reliable, durable and technologically compatible with other components in SOFCs. Additionally, it operates effectively at temperatures higher than 850 °C. However, among the more promising candidate materials is La_1−X_Sr_X_CoO_3_, which belongs to a category of oxides known as mixed ionic and electronic conductors (MIECs). The La_1−X_Sr_X_CoO_3_ has partially been substituted with iron into La0.6Sr0.4Co0.2Fe0.8O3−δ and is generally considered the material of choice for the next generation of low temperature SOFCs. Barium (Ba) is often part of barium strontium cobaltite (BSCF) and barium cerate (BC), which are materials used in SOFC cathodes and proton-conducting electrolytes, respectively. BSCF shows high oxygen permeability and catalytic activity, while BC is known for good protonic conductivity. But Ba-based materials seem to be rather instable. The prominence of Sr in the co-occurrence is because Sr is widely used as a dopant in perovskite-based materials.

Nickel (Ni), frequently paired with yttria-stabilized zirconia (YSZ), forms a composite material used in SOFC anodes, crucial for fuel oxidation processes. As expected, Zr and Y have a very high co-occurrence, reflecting their common usage in zirconia-based electrolytes. Yttrium, scandium, or magnesium are often added to stabilize the zirconia structure and enhance its conductivity. Additionally, the appearance of cerium (Ce) and gadolinium (Gd) points to the growing interest in doped ceria-based materials, such as gadolinium-doped ceria (GDC), which offer enhanced ionic conductivity at intermediate temperatures. Samarium (Sm) and neodymium (Nd) indicate the exploration of alternative materials for improving the performance and durability of SOFCs under various operational conditions. Samarium is used in Sm-doped ceria (SDC) as an electrolyte material or as a catalyst support in SOFCs due to its high oxygen ion conductivity and lower operating temperatures compared to YSZ.

Chromium (Cr) is used in interconnect materials, such as chromium-based alloys, due to their high-temperature oxidation resistance and electrical conductivity.

## 5. Global Distribution and Extraction of Key Elements

Based on the 2020 Critical Raw Materials list, some of the materials necessary for manufacturing SOFCs and SOECs are essential to the European Union’s economy [16].

**Figure 8:**
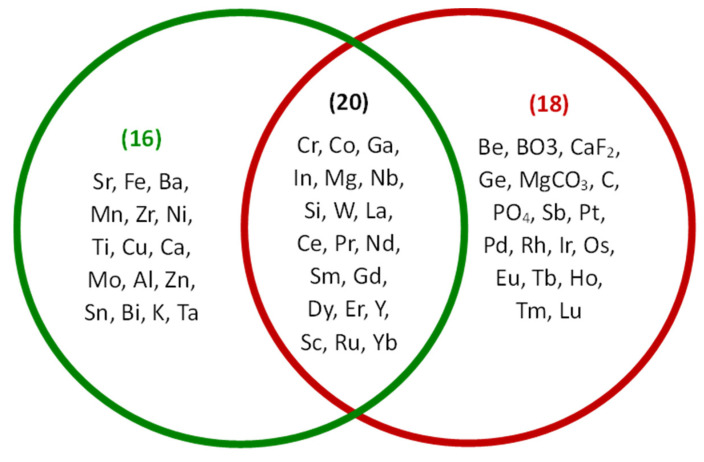
The Venn diagram is classifying the chemical elements into two categories: critical raw materials, in the red circle, and materials commonly used in SOFCs and SOECs, in the green circle, with an intersection indicating elements that overlap both categories.

In Figure 8, the Venn diagram categorizes elements into two primary groups: chemical elements in SOFC and SOEC materials and chemical elements in critical raw materials. These are considered crucial for various industries, including electronics, energy, and transportation, because of their unique properties and limited availability. The overlap between the two groups suggests that certain elements are essential for both SOFC and SOEC applications and they are also classified as critical raw materials. This highlights the potential challenges of securing a stable supply for these elements, as their demand from multiple industries could lead to competition and price fluctuations.

**Figure 9:**
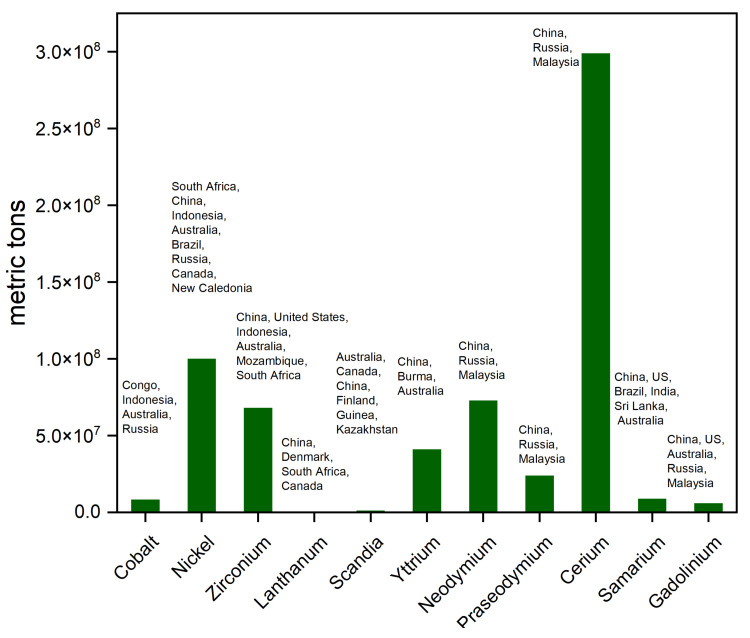
Visually represents the global distribution of key elements across different countries.

In Figure 9, the analysis is extended based on regional aspect. Each country is linked to specific elements, illustrating the presence of these nations in the extraction/production of particular resources [36,37]. The scale helps to show which countries dominate the production or extraction of these materials, allowing easy comparison between countries and materials. The elements listed are shown on the *x*-axis, while the *y*-axis, numerical, indicates the presence of these elements within the countries in metric tons. The chart uses a vertical bars scheme to distinguish between different countries and their associations with particular elements.

**Figure 10:**
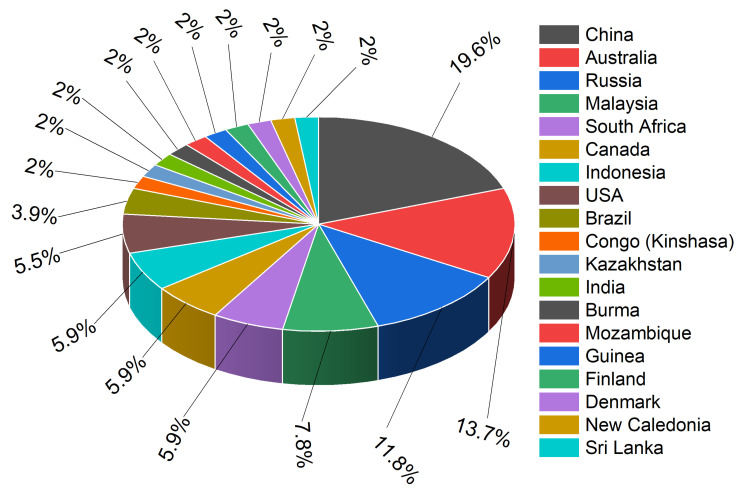
Comparative representation of the global distribution of key elements across different countries.

In Figure 10, the pie chart visually represents the distribution of a specific resource across various countries. Each slice of the pie corresponds to a country, and its size indicates the relative proportion of the resource found in that country.

The majority of the resource comes from China, establishing it as a key player in the global market for this commodity. This dominance may influence the global supply chain, trade policies, and pricing.

Lanthanum, found abundantly in monazite and bastnäsite, shares similar crustal prevalence with cobalt and is sourced mainly from China, Australia and Russia, reflecting the global distribution of rare earth elements [36,37,38,39,40]. The Democratic Republic of Congo is the primary source of cobalt production, with China and Zambia also contributing significantly [36,37,41,42]. Political instability in the Democratic Republic of Congo places a substantial risk to the global cobalt supply. The international supply of nickel has been significantly impacted by recent export-related policies put in place by the Indonesian government. China and South Africa are the primary suppliers of zirconium. China dominates the production of lanthanum, although the United States and Guinea also contribute a significant portion. China is the sole producer of scandia, highlighting a potential supply bottleneck for this critical material. China, Russia, and Malaysia are the main producers of yttrium. Neodymium, praseodymium, cerium, samarium, and gadolinium are primarily produced in China, with some contributions from the United States, Australia, and Malaysia [36,37,38,39,40].

Countries that lack domestic sources of these elements must rely on imports, making them economically dependent on exporting countries. Countries that dominate the production of certain elements have significant control over the global supply chain. This can lead to geopolitical leverage and influence over other countries that depend on these resources. Studies that explore alternative materials that are either more abundant or less expensive need to be conducted. For example, replacing cobalt in cathode materials or reducing the amount of yttrium in the electrolyte can lower costs while maintaining performance.

At the same time, the transition to renewable energy technologies, which rely heavily on many of the elements depicted in this chart, is driving innovation in extraction techniques, recycling processes, and the development of alternative materials. The global competition for control over these resources will continue to shape the economic and political landscape in the years to come, with far-reaching implications for industries, governments, and consumers alike.

**Figure 11:**
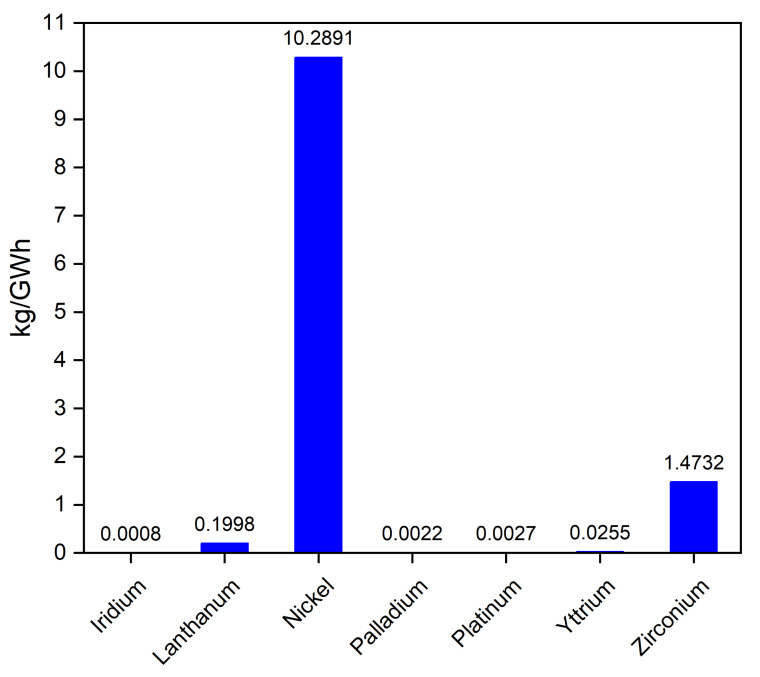
The estimated demand for critical minerals required in electrolyzers and fuel cells, measured in kilograms per gigawatt-hour (kg/GWh).

The graph in Figure 11 reflects the distribution of mineral demands for fuel cells and electrolyzers today, with nickel leading by a significant margin [43].

Nickel has the highest estimated levelized demand by a significant margin, suggesting it is the most critical mineral for electrolyzer and fuel cell production.

Lanthanum and zirconium also have relatively high demand, indicating their importance in these technologies. Other rare earth elements like yttrium and iridium have much lower demand, suggesting they might be less critical or have alternative materials that can be substituted. Platinum and palladium, while in low demand per unit (kg per GWh), are expensive, scarce and essential for these applications [44,45].

## 6. Aspects of Recycling for Materials in SOFCs and SOECs

Recycling is related to its economic value. A significant factor to take into account is the level of effort required for the recycling of specific materials. Certain materials in SOFCs hold more substantial intrinsic value compared to others. The specific cost is associated with extracting, refining, and processing together with location and availability of the material. In particular, the economics of sealant recycling does not make sense because the costs outweigh the benefits, and their recycling is not economically feasible [46,47].

The oxides’ quantities of sealant, such as silicates, aluminates, barium oxide, and calcium oxide, are not critical materials. Sealants typically encounter several forms of degradation, including mechanical failure and leakage, corrosion, and poisoning.

The first step would be disassembling of the stack, mechanically torn apart from each other in a systematic manner, followed by the procedures of selective collection of the metallic and ceramic components [48,49].

The SOFC and SOEC unit should be decommissioned through a controlled shutdown process, ensuring safe disconnection from external power, cooling systems and fuel sources. Subsequently, the stack should be cooled down to a safe operating temperature to prevent thermal shock and facilitate safe handling of components. Because the stack consists of repeating units of fuel cells, the fixtures holding the stack together should be removed. Unstacking is necessary since the individual cell layers comprising the anode–electrolyte–cathode assemblies are taken apart. Next is to separate components by their material types, such as metals, ceramics, and electrolytes.

It was experimentally shown that decoating the oxygen side by ultrasonic decoating is effective. This technique was used as an alternative to the manual mechanical and hydrometallurgical processes, and it appears that it could be automated without the use of environmentally hazardous substances [50].

Another important factor to consider is the long-term durability of the materials used in SOFC and SOEC components. Due to the exposure under a variety of factors simultaneously, during the working schedule, materials in the SOFC and SOEC components undergo complicated degradation processes, changing their physical, chemical and structural properties leading to stack degradation [51,52]. The main degradation mechanisms in the SOFCs/SOECs are phase transition, impurities, and dopant diffusion and mechanical failures due to the difference in TECs of cathode/electrolyte or anode/electrolyte [53,54,55,56,57,58,59,60]. This suggests it would be important to take into account the potential for material contamination when designing recycling processes for SOFC and SOEC component materials.

Following these conditions, the metal or ceramic materials require assessment on the basis of the composition and structure of the respective material. For instance, the materials can undergo examination by X-ray diffraction (XRD), a versatile non-destructive analytical technique to analyze physical properties such as phase composition and crystal structure, elemental analysis such as X-ray fluorescence (XRF), non-destructive analytical technique used to determine the elemental composition of materials, and microscopy techniques.

The degradation mechanisms of cathodes can be due to poisoning by Cr, S, CO_2_, humidity, microstructural deformation and chemical and thermal strains [61,62,63,64,65,66,67]. Cr poisoning can happen in two potential ways for SOFC cathodes; the chemical mechanism involves volatile Cr species reacting directly with cathode materials regardless of the electrochemical reaction at TPBs [50,53,54,55,68,69]. Another mechanism is the electrochemical mechanism, where high-valence volatile Cr species are electrochemically reduced to Cr_2_O_3_ or other low-valance Cr species, and deposited on TPB areas [61,70].

The oxidation of Ni-based anodes and the resulting volume change is probably the most crucial example of chemical stress [71]. The infiltration of undesired oxygen into the anode, whether stemming from system leaks or unregulated fuel utilization, results in irreversible expansion of nickel-based anodes [72]. This mismatch introduces the crack initiation or delamination, which eventually leads to mechanical failure. Generally, anode degradation mechanisms include microstructural changes, coking and poisoning, and delamination. Although delamination occurs less often in the anode compared to the cathode, it is not completely eliminated. The most common microstructural changes in Ni-based anodes are Ni coarsening, Ni migration, and Ni depletion, which are somehow connected to each other [73,74,75,76]. The process of nickel coarsening is recognized as the most harmful degradation mechanism affecting SOFC and SOEC anode electrodes. The main cause is surface diffusion at the interface, typically associated with a mechanism related to Ostwald ripening [72]. Ostwald ripening is the process by which the small particles within a solution dissolve and adhere to larger particles, aiming to attain a thermodynamically more stable condition characterized by the minimization of the surface-to-area ratio [77,78]. Microstructural changes occurring in the Ni/YSZ electrode during long-term operation have a strong impact on the performance loss of the cell. The Ni/YSZ electrode microstructure of two nominally identical cells tested for 1000 h, one in SOFC mode and the other in SOEC mode, was examined. It was observed that a significant Ni migration away from the electrode/electrolyte interface was detected only for the SOEC case [79].

As noted earlier, one of the benefits of SOFCs/SOECs is their capacity for fuel flexibility, allowing them to perform internal reforming of hydrocarbon fuels at high operating temperatures. Nevertheless, there is a possibility of anode coking due to the ongoing reactions of the carbon monoxide generated during the reforming process, which is specifically related to the Boudouard reaction [80,81].

2CO(g) ⇌ CO_2_(g) + C(s) (5)

This CO reacts with H_2_ as well and results in more carbon formation. The rate of coking is affected by various factors, such as the steam-to-carbon ratio, anode composition, operating temperature, and current density [82,83,84]. For example, the carbon deposition exhibits an inverse correlation with both the steam-to-carbon ratio and the applied current density. Under the same operating conditions, carbon deposition was found to decrease with increasing temperature on Ni/ScSZ cermet electrodes, but it increased with increasing temperature on Ni/YSZ cermet anodes [84]. Siloxanes, which are contaminants found in biogas, can cause the formation of solid SiO_2_, both within the surface and within the porous structure, during operation [85].

The phenomenon of anode coking is characterized by a surface coating that blocks the triple phase boundaries (TPBs) and gas channels, which contributes to electrochemical and mechanical degradation. As carbon deposition escalates, it generates substantial pressure that could ultimately lead to anode rupture.

Apart from coking, hydrocarbon gaseous fuels are composed of different contaminants, including sulfur (S), phosphorous (P), arsenic (As), selenium (Se), chlorine (Cl), and antimony (Sb), that may interfere with the anode and degrade the performance and stability of the SOFC [72,83], as well as the nature and quantity of these elements present in the hydrocarbon fuel.

The most notable phenomenon is the electrolyte phase transformation, for instance, from cubic to tetragonal zirconia, which strongly depends on the Y_2_O_3_ concentration in ZrO_2_ [74,86,87]. The chemical interactions between the electrolyte and especially the cathode, give rise to the formation interface and insulating secondary phases: LSCF/YSZ, GDC/YSZ and LSCF/GDC systems. The chemical reactions occurring at the electrolyte–anode side are not that harsh compared to those at the electrolyte–cathode side. Ni-YSZ, being a widespread anode material, does not encounter issues with the YSZ electrolyte.

The most common interconnects are LaCrO_3_-based interconnects, which are p-type semiconductor oxides that favor phenomena of Cr vaporization and mechanical failures [88]. The release of chromium vapor from interconnects results in chromium contamination of the cathodes, leading to degradation process in SOFCs/SOECs. This phenomenon produces substantial reduction in electrical conductivity by obstructing the active triple-phase boundary sites of the electrode. Furthermore, this Cr vaporization induces the Cr depletion in the interconnect, and this depletion below a specific threshold threatens its mechanical strength and structural integrity through the oxidation break-away.

The formation of toxic nickel oxide and chromium oxide are known to be harmful to human health and the environment [89]. Consequently, it will be necessary to devise automated disassembly methods in the future.

The third step aims to extract valuable materials from the recycled SOFC or SOEC components in order to reuse the individual parts/materials. This step could be subdivided into (i) direct re-use, with/without refurbishing, within new SOFCs and SOECs; (ii) direct reuse in new SOFC and SOEC components after procedures such as cleaning, grinding, solving, enrichment, etc.; (iii) indirect re-use in other valuable products such as steels with less mechanical and chemical requirements, ceramics within refractory materials, etc.; or (iv) safe disposal which should of course be avoided if possible.

Closed-loop recycling is characterized as a procedure in which waste materials are gathered, recycled, and subsequently reutilized in the production of the identical product from which they originated [90,91]. In closed-loop recycling, manufacturing processes are typically engineered, taking into consideration the recycling process. This closed-loop approach ensures that materials are reused, minimizing waste and reducing the overall environmental impact.

Repurposing can be for other applications if they cannot be directly reused in SOFCs/SOECs. This could involve grinding the components into a powder form and using them as fillers in the construction industry or as additives in other manufacturing processes.

Major difficulties in recycling SOFC and SOEC materials evolve around the development of effective separation technologies, the handling of toxic substances, and the optimization of recycling operations. It is important to note that recycling SOFC and SOEC components require careful handling and processing to ensure the safety of workers and the environment, following proper guidelines and regulations for recycling these materials [79].

## 7. Aspects of Isolating and Extracting Component Materials in SOFCs and SOECs

Numerous studies have been carried out on the separation of rare earth elements using different media and extraction agents [92,93,94,95]. Although these studies and published works on the recovery of rare earth elements are numerous, research specifically on the elements coming from the materials of the SOFC and SOEC components remains relatively unexplored. For instance, many studies were focused on the importance of finding ways for recycling rare earth elements from permanent magnets [96]. Chemical processes like leaching and solvent extraction are utilized to selectively dissolve or extract specific components or metals from the SOFC and SOEC materials.

Leaching is a technique extensively used in extractive metallurgy. This technique involves chemicals for ore processing. In this way, valuable metals are converted into soluble salts while leaving impurities behind as insoluble materials. The soluble salts are separated and further processed to obtain pure metals, and the remaining byproduct of mining is called the tailings. Acid leaching is useful for dissolving and recovering metals such as nickel or cobalt from the anode or cathode layers. Solvent extraction techniques can also be used for selective separation and recovery of metals [97,98].

Electrowinning is another technique that can be used for this purpose [99,100]. This technique is an electrochemical process used to extract metal ions from aqueous solutions. The basic principle relies on using an electric current to induce a reduction reaction at the cathode. This causes the metal ions to precipitate in a solid form.

This technique can be used to recover metals from the leachate obtained during the recycling process. By applying an electric current, the desired metal ions can be selectively plated onto an electrode, allowing for their recovery in a pure form.

Solvent extraction has demonstrated its effectiveness in separating and extracting rare earth metals according to some studies [86,87]. Despite their maturity level, certain drawbacks, such as the excessive consumption of solvents and extractants, high energy consumption, and the generation of large amounts of toxic gasses, render the method costly and environmentally detrimental [101,102,103,104].

Typically using traditional hydrometallurgical and pyro-hydrometallurgical methods [105], EoL technologies primarily aim to recover PGMs used as electrocatalysts [106]. However, innovative processes can be implemented to recover not only PGMs but also other important materials like carbon support and ionomers from fuel cell stacks [107].

The Pt catalyst for PEMFC was studied [108]. In their study, the recycling of Pt/C was by a hydrometallurgical approach using ion exchange or solvent extraction and polyol synthesis and the recycling of Pt/C by a carbon-based extraction process. The transmission electron microscopy morphological characteristics of the Pt/C recycled via the ion exchange resin alternative are closely alike to the benchmark catalyst, and the Pt nanoparticles obtained by the carbon-based extraction process are homogenously dispersed on the carbon support [108].

The advancement of battery material recovery can serve as a model for other recycling initiatives. In recent years, recycling of battery materials increased for the same reason as the demand for metal. In the laboratory, lithium, manganese, cobalt, and nickel were recovered by hydrometallurgical techniques from the leaching solution of spent lithium-ion batteries [109].

A water-based deep eutectic solvent composed of choline chloride and lactic acid was proposed for Co and Li recovery from spent LiCoO_2_ cathode materials [110]. The author’s focus was on developing a method employing mild conditions and promoting sustainable development of spent LIBs in the recycling industry.

Likewise, conventional methods have been tried for the recovery of rare earth elements from the permanent REE magnets, nickel metal-hydride batteries, lamp phosphors, cathode ray tubes, glass polishing powders, fluid cracking catalysts, and optical glass [111].

A hydrometallurgical process was developed for rare earths extraction from spent optical glass [112,113]. The process consists of (a) conversion of amorphous rare earths borosilicates to insoluble rare earths hydroxides by hot aqueous sodium hydroxide with high concentration; and (b) leaching of the rare earth hydroxides using hot hydrochloric acid to obtain a mixture of rare earths chlorides in aqueous solution.

The extraction of rare earth metals from technological solutions obtained during the processing of apatite concentrates is a complex process involving several steps. The kinetic features of solid-phase extraction of samarium (III) and gadolinium (III) from simulated industrial phosphoric acid solutions was studied and the limiting stage of the extraction process on a solid extractant can be considered a chemical reaction between ions of rare earth metals and functional groups of the extractant [114].

La_2_O_3_, Y_2_O_3_ and their mixture were electrolytically decomposed using Ir wires, confirming the electrolytic nature for these melts and the ability to extract rare earth elements [115]. Oxygen gas evolution was observed at the anode and La and Y were observed as co-deposits for the La_2_O_3_-Y_2_O_3_ mixtures investigated.

An all-atom molecular dynamics simulation to understand the separation behavior of the gadolinium ions (Gd^3+^) and its nitrates (NO_3_^−^) from the aqueous wastewater using directional solvent extraction with decanoic acid as an extractant was analyzed and it was observed that a decrease in H_2_O density was due to the aggregation of Gd^3+^ and NO^3−^ [116].

Hydrothermal recovery of YSZ from EoL of SOFCs showed the efficacy of the investigated Ni extraction approach leading to a recovered YSZ phase with negligible nickel residues and appropriate structural, crystallinity, and morphological features, as well as average particle size [117].

A multi-step milling/sieving process was investigated for the recovery of NiO-YSZ composite powders from waste half-cells derived from SOFC production [118].

At the laboratory scale, more than 92% of nickel oxide and YSZ were recovered. YSZ was recovered as a 4YSZ powder of micrometer range size, with less than 0.5 At% Si impurities, using nitric acid leaching. The raw air electrode material is produced through a straightforward mechanical scratching process. The material is soaked in a solution of HNO_3_ (65 wt.%), in order to dissolve the nickel. The solution becomes green, in accordance with the presence of Ni^2+^ ions, whereas a white sediment appears at the bottom of the container. A centrifugation process was used to separate the green solution and sediment [119].

Another recovery strategy involved recycling waste materials generated during SOFC manufacturing. To recover waste materials generated during the production of NiO-YSZ anodes, two methods were used: tape casting coupled with isostatic pressing and extrusion [120]. Additives were removed through a burnout process, and subsequent milling yielded powders with properties similar to commercial powders.

Validating efficient and scalable recycling methods, yet environmentally friendly, to recover essential raw materials remains an unaddressed challenge for SOFCs and SOECs.

## 8. Concluding Remarks

The present work highlights a substantial gap in the knowledge of recycling procedures aimed at recovering component materials from the end-of-life of SOFC and SOEC components, with the goal of reusing these materials in agreement with the circular economy principles, where materials are reused, recycled or regenerated. This is essential for creating sustainable energy systems. SOFCs and SOECs stand out as promising clean energy technologies, but they face ongoing challenges in securing a reliable and sustainable supply of critical materials.

With the exception of a few published papers, there is a lack of research focusing on tailored recovery methods that address the unique requirements and challenges posed by all components of SOFCs and SOECs technology.

A feasible answer to these material challenges could be recycling. Implementing closed-loop material flows can lead to a more sustainable future. Yet the methods demonstrated thus far have been limited to laboratory conditions with controlled conditions. For broader implementation, these methods must be scaled up and tested in facilities to simulate real-world scenarios. In addition, detailed life-cycle assessments and economic analyses are needed to evaluate the benefits of recycling processes. The overall objective of recycling materials from SOFC and SOEC-type system components is to recover valuable materials in an efficient and cost-effective manner. Some materials are more valuable because of their quantity since they are abundant in the SOFC and SOEC stack or because of their production cost or difficulty in obtaining them. However, the degradation of the component materials during the operation of SOFC and SOEC systems represents another challenge. As a result of their chemical degradation, for example, these materials may lose their initial functional properties. Consequently, those materials can no longer be used for the same initial purpose in the manufacturing of SOFC and SOEC-type systems.

Waste can undoubtedly be reduced by promoting efficient recycling processes. This approach will consequently reduce the impact on the environment and help to conserve valuable resources.

More research and innovation initiatives are crucial to tackle the current challenges and unlock the full potential of these technologies. Clearly, research initiatives focusing on the development of cutting-edge materials and innovative recycling technologies are needed. Large-scale recycling of SOFCs and SOECs should surpass the limitation for the absence of an efficient and selective collection system for their material components. Promising avenues for future investigations would be material synthesis routes with a focus on reuse. One promising area of research is additive manufacturing for component repair and modular design in SOFCs and SOECs. This approach could significantly enhance the sustainability of these systems by extending their operational life and reducing the need for entirely new components. Experiments could also focus on extending the lifespan of critical parts by regenerative treatments to reduce the need for entirely new materials.

The integration of circular economy concepts into energy infrastructure, particularly in SOFC and SOEC systems, is crucial to promote long-term sustainability. This will contribute to reduce dependence on finite resources and significantly reduce the carbon footprint associated with material extraction, production and end-of-life disposal.

## Data Availability

Not applicable.
